# Functional role of Galectin-9 in nucleic acid trafficking and transcription post-electrotransfection

**DOI:** 10.1093/narmme/ugag020

**Published:** 2026-03-26

**Authors:** Yifei Wang, Justin Sylvers, Fan Yuan

**Affiliations:** Department of Biomedical Engineering, Duke University, Durham, NC 27708, United States; Department of Biomedical Engineering, Duke University, Durham, NC 27708, United States; Department of Biomedical Engineering, Duke University, Durham, NC 27708, United States

## Abstract

Galectin-9 (Gal9) is known for its cytoplasmic roles in vesicular damage responses, yet its active functional roles in gene delivery remain unclear. Here, we investigated Gal9 dynamics in electrotransfection (ET), a non-viral gene delivery method widely used for plasmid DNA (pDNA) and mRNA transfection. Confocal imaging revealed unexpected nuclear accumulation of Gal9 after pDNA ET. Gal9 colocalized with SC35, a biomarker of nuclear speckles, and immunogold TEM confirmed its enrichment within those electron-dense, subnuclear domains following pDNA ET. Functionally, Gal9 knockdown markedly reduced ET efficiency and reporter gene expression, whereas overexpression enhanced both. The timing and the extent of Gal9–pDNA association varied with delivery method and cargo type, being prominent for DNA but minimal for mRNA. Across different cell types, endogenous Gal9 expression correlated with ET efficiency. These findings revealed previously unrecognized, active functional roles of Gal9 in organizing exogenous DNA within transcriptionally active regions of the nucleus and regulating transgene expression following their non-viral delivery.

## Graphical Abstract

**Graphical Abstract:**
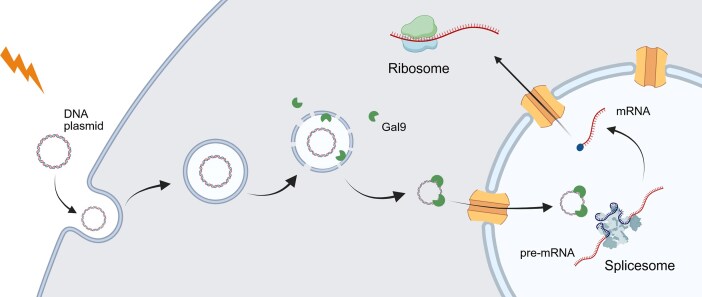

## Introduction

Galectin-9 (Gal9) is a member of the galectin family of β-galactoside–binding proteins that function in a variety of cellular processes, including immune regulation, cell adhesion, apoptosis, and vesicular trafficking [1, 2]. Structurally, Gal9 is composed of two carbohydrate recognition domains connected by a flexible linker, allowing it to recognize and bind to glycosylated ligands on damaged intracellular membranes [3]. While originally studied for its immunomodulatory roles, such as inducing apoptosis in T cells and contributing to T cell exhaustion in chronic viral infections and cancer [4–6], Gal9 has more recently been recognized for its involvement in vesicle integrity monitoring and immune signaling. In particular, it is robustly recruited to damaged endosomes and lysosomes, where it acts as a sensor of membrane damage and coordinates downstream repair responses [3, 7]. This feature has made Gal9, along with other galectins, a useful reporter for vesicle membrane damage triggered by cargo escape of various delivery systems [8].

While the cytoplasmic and vesicular functions of Gal9 are well established, its potential roles beyond the vesicular compartment remain largely unexplored. Although nuclear localization of Gal9 has been occasionally observed, its biological significance and possible involvement in nucleic acid–related processes have not been investigated. Understanding whether Gal9 actively participates in gene delivery-related intracellular and intranuclear events could substantially expand current knowledge of its cellular functions and provide new insight into how this protein influences cargo transport, including nucleic acid delivery.

Electrotransfection (ET) provides a powerful platform to study intracellular nucleic acid trafficking and the involvement of cellular proteins in the transport processes. As a non-viral gene delivery method, ET introduces plasmid DNA (pDNA) into mammalian cells through electric pulse application, triggering pDNA cellular uptake primarily through endocytic pathways [9, 10]. After internalization, pDNA undergoes complex intracellular trafficking, including endosomal–lysosomal transport and eventual nuclear entry, before transgene expression occurs [9–12]. Understanding how host cellular proteins, such as Gal9, are involved in these steps is essential for improving ET performance and advancing non-viral delivery strategies. Given Gal9’s established roles in vesicle dynamics and vesicular membrane damage sensing, we hypothesized that it may participate in one or more of these intracellular processes that govern pDNA trafficking and nuclear delivery.

To explore the potential involvement of Gal9 in intracellular events after ET, we examined its spatial distribution in cells following pDNA delivery. Unexpectedly, Gal9 was detected not only in the cytoplasm but also within the nucleus post-pDNA ET. This finding prompted us to investigate whether Gal9 exerts additional nuclear functions during and after ET. Our data reveal that Gal9 colocalizes with transcriptionally active hubs in the nucleus and functionally modulates transgene expression from electrotransfected plasmids, uncovering a previously unrecognized nuclear role for Gal9 in nucleic acid trafficking and transcription during non-viral gene delivery.

## Material and methods

### Cell lines and culture

C2C12, a mouse myoblast cell line, was obtained from the Duke University Cell Culture Facility (CCF). Cells were cultured in DMEM (Gibco), supplemented with 10% (v/v) bovine calf serum (Cytiva) and 1% penicillin–streptomycin (Gibco). Cultures were maintained at 37°C in a humidified atmosphere with 5% CO_2_ and passaged every 2–3 days.

### Electrotransfection

The C2C12 cells at ∼70–80% confluency were detached from culture plates by treatment with 0.25% Trypsin-EDTA (Gibco). Collected cells were washed once with DPBS (Gibco). For each electrotransfection, 1.0 × 10^6^ cells were resuspended in 100 μL of pulsing buffer, Opti-MEM™ GlutaMAX™ (Gibco), consisting of pDNA, minicircle DNA, or mRNA at indicated concentrations. The cell mixture was transferred to an electroporation cuvette with a 4-mm gap (Genesee). Cells were pulsed at a condition of 250 V, 10 ms duration, 2 pulses with a 10-s interval using the BTX ECM830 Square Wave Electroporation System (Harvard Apparatus). Immediately after pulsing, pre-warmed (37°C) DMEM full medium was added to the cuvettes. The cells were then seeded in either six-well plates or #1.5 glass-bottom dishes (Cellvis). For cell synchronization, mimosine was added to the complete medium at a final concentration of 400 μM. Cells were pretreated with mimosine for 24 h prior to electrotransfection of plasmid DNA. Afterward, cells were incubated with EdU for 16 h. All cells were maintained at 37°C for the indicated time periods prior to flow cytometry analysis or fixed in 4% PFA for 10 min at room temperature for fluorescence microscopy.

### Generation of Gal9-expressing stable cell line

C2C12 cells were transfected with a plasmid encoding Gal9-GFP using the Lipofectamine 3000 kit (Invitrogen), following the manufacturer’s protocol. Briefly, cells were seeded in six-well plates at 70–90% confluency. Lipofectamine 3000 reagent and plasmid DNA (pDNA) were diluted in Opti-MEM™ Reduced Serum Medium (Gibco), after which the P3000™ reagent was added to the diluted pDNA solution. The P3000–DNA mixture was then combined with the diluted Lipofectamine 3000 reagent at a 1:1 ratio and incubated for 10–15 min to allow complex formation. The resulting DNA–lipid complexes were added to the cells for transfection.

After 24–48 h of incubation, transfected cells were transferred to antibiotic-containing complete medium for selection. To isolate single clones, cells were serially diluted and seeded at densities ranging from 100 to 10⁵ cells per plate. The cultures were maintained under selection pressure for 14–20 days, during which single colonies were allowed to expand. Fluorescent colonies were identified using fluorescence microscopy and individually picked. Colonies were first transferred to 24-well plates, then progressively expanded to six-well plates and finally to 10 cm dishes as they reached confluency. Approximately one million cells from each clone were analyzed by flow cytometry to determine transgene expression levels. Clones exhibiting strong fluorescence and expression level were selected and cryopreserved for future use.

### SC35 antibody staining

C2C12 cells grown on glass petri dishes were briefly washed with DPBS, then fixed and permeabilized with 4% formaldehyde and 0.2% Triton X-100 in DPBS for 10 min at room temperature, followed by incubation in pre-chilled acetone at –20°C for 5 min. After fixation, cells were washed three times with DPBS. SC35 staining was performed using Alexa Fluor 568-conjugated SC35 antibody (Abcam), diluted 1:200 in DPBS, and incubated with the cells for 60 min at room temperature in a humidified chamber. Following incubation, cells were washed three times with DPBS, stained with Hoechst (10 μg/mL) for 3 min, and prepared for fluorescence microscopy.

### Immuno-TEM

Six hours post-electrotransfection, C2C12 cells pulsed with or without pDNA were fixed in 4% PFA in PBS for 1 h at room temperature, washed, and stored at 4°C. After dehydration through a graded ethanol series, samples were embedded in resin, and ultrathin sections were collected on nickel grids. Sections were blocked with 5% normal goat serum (NGS) in PBS containing 0.1% BSA and 0.05% Tween-20 for 30 min, followed by overnight incubation at 4°C with a primary antibody against Galectin-9 diluted in blocking buffer. After washing, grids were incubated with a 10 nm gold-conjugated secondary antibody for 1 h at room temperature. Sections were then washed, post-stained with uranyl acetate and lead citrate, and imaged using a transmission electron microscope (JEOL JEM 1230) at 80–100 kV.

### siRNA-mediated knockdown of Gal9 proteins

Small interfering RNA (siRNA) targeting Lgals9 (Assay ID: s69187) was designed against mouse Lgals9 transcripts (NM_010708.2, NM_001159301.1), covering multiple variants (ThermoFisher). A non-targeting siRNA (Silencer Select Negative Control No. 1) served as a negative control (Catalog No. 4390843, ThermoFisher). This control has no significant sequence homology to human, mouse, or rat genes and shares similar chemical modifications with targeting siRNA to control for non-specific effects of transfection and siRNA treatment. 

C2C12 cells were transfected with siRNA targeting Galectin-9 (Gal9) using Lipofectamine™ RNAiMAX Transfection Reagent (ThermoFisher) following the manufacturer’s protocol. Briefly, cells were seeded in complete DMEM growth medium to reach 60–80% confluency on the day of transfection. For each well of a six-well plate, siRNA (50 pmol) and Lipofectamine RNAiMAX reagent were diluted separately in Opti-MEM™ Reduced Serum Medium (Gibco), then combined and incubated for 5 min at room temperature to allow complex formation. The siRNA–lipid complexes were added directly to the cells, which were then incubated under standard culture conditions (37 °C, 5% CO₂). Knockdown efficiency was assessed 24–72 h post-transfection by western blot. A non-targeting siRNA was used as a negative control and included in all downstream analyses.

### Western blot analysis for Gal9

Western blot analysis was performed to assess Gal9 protein levels following siRNA-mediated knockdown, Gal9 overexpression, and to compare basal expression across different cell lines. At 24–72 h post-transfection, cells were collected and lysed in a buffer containing 750 mM NaCl, 5 × TE buffer, 1% Triton X-100, and 1 × protease inhibitor cocktail. Total protein concentrations were quantified using the BCA Protein Assay Kit (ThermoFisher). Equal amounts of protein from each sample were separated on NuPAGE™ 4–12% Bis-Tris gels (ThermoFisher) and transferred onto nitrocellulose membranes. Gal9 proteins were detected using a primary anti-Galectin-9 antibody and an HRP-conjugated secondary antibody (Invitrogen). Chemiluminescent signals were developed using the SuperSignal™ West Femto Maximum Sensitivity Substrate (ThermoFisher) and imaged with the iBright Imaging System (ThermoFisher). β-Actin was used as an internal control across measured samples. Image J was used as the analytic tool to quantify the protein bands.

### *In situ* hybridization with DNA probes

Hybridization was used to detect the non-labeled pEGFP plasmid electrotransfected into C2C12 cells. Cells cultured on glass-bottom Petri dishes were washed once with DPBS and fixed with 4% paraformaldehyde (PFA) for 10 min at room temperature. Hybridization was performed using the RNAscope Multiplex Fluorescent Reagent Kit v2 (ACD), with modifications to detect plasmid DNA. Briefly, fixed cells were first incubated with hydrogen peroxide for 10 min at room temperature, followed by washing with ultrapure distilled water. Cells were then treated with RNA digestion buffer (25 μg/mL RNase A in 1 × TBST) for 30 min at 37°C, followed by two washes with DPBS. Next, samples were treated with Protease III for 10 min at room temperature and washed twice with DPBS. DNA denaturation was performed by incubating the cells in pre-warmed denaturation buffer (75% formamide in 2 × SSC) for 5 min at 75°C, followed by a DPBS wash. Cells were then incubated with the primary detection probe (Probe-EGFP-O4-Sense-C1) for 4 h at 40°C. Following hybridization, cells were sequentially incubated with two hybridization amplifiers for 30 min each, followed by a third amplifier for 15 min, all at 40°C. Fluorescent signal development was performed by incubation with HRP for 15 min, followed by Opal 690 (Akoya), diluted 1:1500 in TSA buffer, for 30 min at 40°C. RNAscope wash buffer was used between each incubation step. Finally, samples were stained with Hoechst (10 μg/μL) for 3 min at room temperature prior to fluorescence microscopy analysis.

### Flow cytometry analysis

To evaluate transgene expression, C2C12 cells electrotransfected with pEGFP were collected at 24 h post-transfection and analyzed using a NovoCyte flow cytometer (Agilent) with NovoExpress software. Prior to analysis, cells were washed with DPBS and resuspended in DMEM full medium. Single-cell populations were gated based on forward and side-scattered light, with gating thresholds established using untransfected cells as controls. EGFP fluorescence was detected using the 488 nm channel. Electrotransfection efficiency was defined as the percentage of EGFP-positive cells within the gated population. Expression level was quantified as the geometric mean of EGFP fluorescence intensity among the EGFP-positive cells.

### Fluorescence microscopy and image analysis

Fluorescence images for pDNA, Gal9, and SC35 analyses were taken using an Andor Dragonfly spinning disk confocal microscope equipped with a × 63 oil objective. Super-resolution imaging was performed using the super-resolution radial fluctuations (SRRF) method implemented in the Andor Dragonfly system. Multiple-channels were used to acquire images for cell samples: Hoechst staining for nuclei with a 405 nm laser and 450/50 nm emission filter, Gal9-GFP signal with a 488 nm laser and 525/50 nm emission filter, SC35 stained by an antibody conjugated with Alex Fluor 568 with a 561 nm laser and 600/50 nm emission filter, and Cy5 or Opal 690 with a 637 nm laser and 700/75 nm emission filter. All images were taken near the central plane of most cell nuclei in the field of view, and out-of-focus nuclei were excluded automatically or manually based on the Hoechst signal.

Image analysis was performed with CellProfiler. An automated analysis previously developed in our lab was used to quantify the pDNA and Gal9 in nucleus [13]. Briefly, nuclei were segmented from the Hoechst channel based on size (150–600 pixels, ∼7–29 μm diameter) and smoothed to exclude objects with jagged edges. Out-of-focus nuclei were removed using a form factor cutoff, with only objects between 0.7 and 1.0 retained for analysis. Each in-focus nucleus was further divided into edge and inner regions using concentric ring partitioning. Plasmid DNA (pDNA) signals in the far-red channel were identified following background subtraction using negative controls and segmented as punctate objects with diameters between 4 and 40 pixels (approximately 190–1900 nm) and fluorescence intensities exceeding a manually defined threshold based on the experimental average. Galectin-9 puncta were identified as discrete objects with diameters between 0.5 and 2 μm and fluorescence intensities exceeding a manually defined threshold, established using untransfected control cells. Colocalization between Galectin-9 and SC35 was scored when the overlapping area between a Gal9 punctum and SC35 signal occupied at least 30% of the Gal9 punctate area. All segmentation parameters and intensity thresholds were held constant across experimental conditions within each analysis set to ensure consistency and comparability.

### Statistical analysis

Statistical analysis was performed using GraphPad Prism software. The Mann–Whitney U test was used to assess statistical significance between two experimental groups; for comparisons among multiple groups, one-way ANOVA was performed, followed by Tukey’s HSD test for pairwise comparisons. Significance levels are indicated as follows: ns, not significant; *P < 0.05; **P < 0.01; ***P < 0.001; ****P < 0.0001. Outliers were excluded using the standard deviation method, in which data points deviating more than two standard deviations from the group mean were removed from analysis.

## Results

### Nuclear localization of Galectin-9 post-pDNA electrotransfection

**Figure 1.**
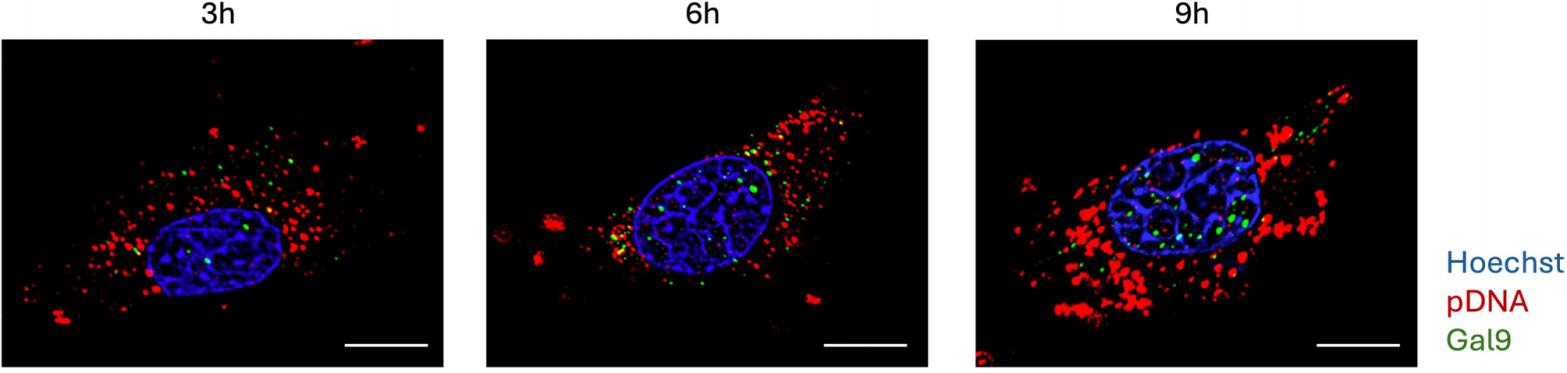
Time-dependent nuclear localization of Galectin-9 and electrotransfected plasmid DNA in super-resolution images. Stably Gal9-GFP–expressing C2C12 cells were electrotransfected with Cy5-labeled plasmid DNA (red) and imaged by confocal fluorescence microscopy at 3, 6, and 9 h post-ET. Hoechst (blue) was used to stain nuclei, and Gal9-GFP (green) was used to monitor Gal9 localization. Scale bars: 10 μm.

To investigate the spatiotemporal dynamics of Gal9 subcellular distribution, stably Gal9-GFP–expressing C2C12 cells were electrotransfected with Cy5-labeled plasmid DNA (pDNA) and analyzed at different time points post-pulsing using confocal fluorescence microscopy. As expected, Gal9 puncta were observed in the cytoplasm, consistent with its established role in endosomal damage responses. Interestingly, we also observed an unexpected localization of Gal9 within the nucleus post-electrotransfection of pDNA. These nuclear Gal9 signals appeared as distinct puncta and were predominantly detected in cells that were positive for Cy5-pDNA, suggesting a potential interaction between Gal9 and the delivered plasmid DNA (Fig. 1). This nuclear accumulation was observed not only for GFP-tagged Gal9 but also for endogenous Galectin-9, as confirmed by confocal microscopy following immunostaining (Supplementary Fig. S1). By contrast, control cells (non-transfected or subjected to electric pulsing in the absence of plasmid DNA) did not exhibit obvious punctate patten of Gal9 accumulation in either the nucleus or the cytoplasm, with Gal9 remaining largely diffuse (Supplementary Fig. S2). These observations indicate that the Gal9 nuclear localization pattern is dependent on plasmid DNA delivery rather than electric pulsing alone.

Importantly, the nuclear localization of Gal9 persisted over time rather than appearing as a transient event immediately after ET. Confocal images captured at 3, 6, and 9 h post-electrotransfection revealed dynamic changes in the intracellular distribution of both Gal9 and pDNA. At 3 h, signals for both Gal9 and pDNA were primarily confined to the cytoplasm, with minimal nuclear presence. By 6 h, increased nuclear localization of both signals was observed, and this trend became more significant at 9 h. At this later time point, Gal9 formed larger and more defined puncta within the nucleus, paralleling an increase in nuclear pDNA accumulation. This temporal pattern suggests that nuclear trafficking, retention, and transcriptional engagement of pDNA may be potentially accompanied and facilitated by the progressive recruitment of Gal9 to the nucleus.

These findings expand the current understanding of Gal9 beyond its canonical function in cytoplasmic endosomal damage responses, indicating a potential novel mechanism by which Gal9 may facilitate nuclear delivery and transcription of plasmid DNA following electrotransfection.

### Galectin-9 nuclear localization is independent of cell division

**Figure 2.**
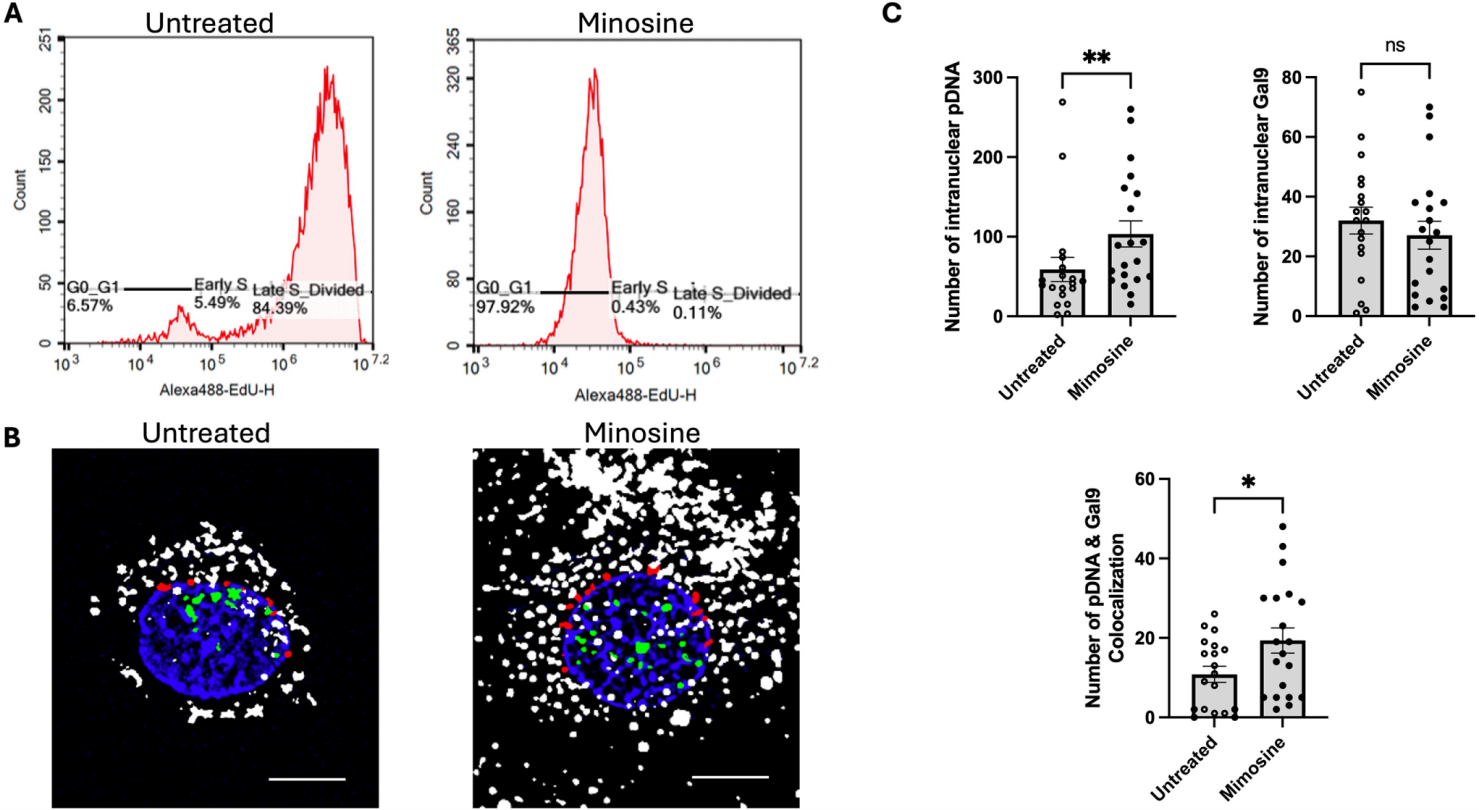
Galectin-9 nuclear localization in untreated and mimosine-treated cells. **(A)** Quantification of cell proliferation with EdU. C2C12 cells were pretreated with or without mimosine for 24 h, followed by electrotransfection of pLuc and incubation with EdU for 16 h. The cells were analyzed with flow cytometry. The histograms of EdU signal were used to quantify the percentages of cells in G0/G1 phase, early S phase, and late S phase. **(B)** Typical super-resolution images of Cy5-pLuc and Gal9-GFP post-ET in untreated and mimosine-treated cells. Hoechst-stained nuclei (blue), Cy5-pDNA signals (white), colocalized Gal9-GFP with pDNA in the inner nucleus (green), and pDNA localized on the nuclear edge (red). Scale bars: 10 μm. **(C)** Quantification of pDNA, Gal9, and pDNA-Gal9 colocalization in untreated and mimosine-treated cells. *n* = 20. mean ± SEM; *P < 0.05, **P < 0.01, ns = not significant (Mann–Whitney U test).

Although some electrotransfected plasmid DNA molecules can access the nucleus through passive inclusion during mitosis, previous findings indicate that nuclear entry of pDNA can also occur independently of cell division. In particular, we have observed that cell cycle-arrested cells tend to exhibit more punctate pDNA signals within the nucleus, suggesting the involvement of active nuclear trafficking mechanisms. To investigate whether Gal9 nuclear localization occurs independently of mitosis and to assess its potential role in nuclear pDNA processing, we arrested C2C12 cells in G1 phase using 400 μM mimosine prior to electrotransfection. EdU incorporation and flow cytometry analysis confirmed successful synchronization, with over 97% of cells retained in G1 phase (Fig. 2A).

Confocal imaging showed that Gal9 nuclear puncta were consistently present in mimosine-treated cells, indicating that Gal9 nuclear localization is independent of passive inclusion during nuclear envelope breakdown and reformation in mitosis (2B). Quantitative image analysis revealed a significant increase in the number of nuclear pDNA puncta in the mimosine-treated group compared to the untreated group (Fig. 2C), consistent with the qualitative confocal observations and our prior findings in cell cycle-arrested COS7 cells [13]. In contrast, the number of nuclear Gal9 puncta did not differ significantly between the two groups, supporting the hypothesis that Gal9 is not passively incorporated into the nucleus through cell division.

In addition, colocalization analysis showed a significant increase in pDNA and Gal9 overlap within the nucleus of mimosine-treated cells. This suggests that although the total number of Gal9 puncta remained constant, a large fraction of these puncta were associated with nuclear pDNA in arrested cells. These findings imply that Gal9 is not only involved in cytoplasmic pDNA trafficking but may also play a vital role in post-entry nuclear activities such as pDNA retention or recruitment to transcriptionally active nuclear compartments.

### Galectin-9 colocalizes with transcriptional hubs in the nucleus

In our previous study, we observed that plasmid DNA delivered by electrotransfection presents in two distinct nuclear patterns: diffuse signals and discrete punctate structures. The latter increased upon mimosine treatment and correlated with enhanced reporter mRNA levels, supporting the hypothesis that punctate signals may represent pDNA enriched in transcriptionally active hubs [13]. Building on this, we investigated whether nuclear-localized Galectin-9 plays a role in facilitating the recruitment of pDNA to transcriptionally active subnuclear compartments.

**Figure 3.**
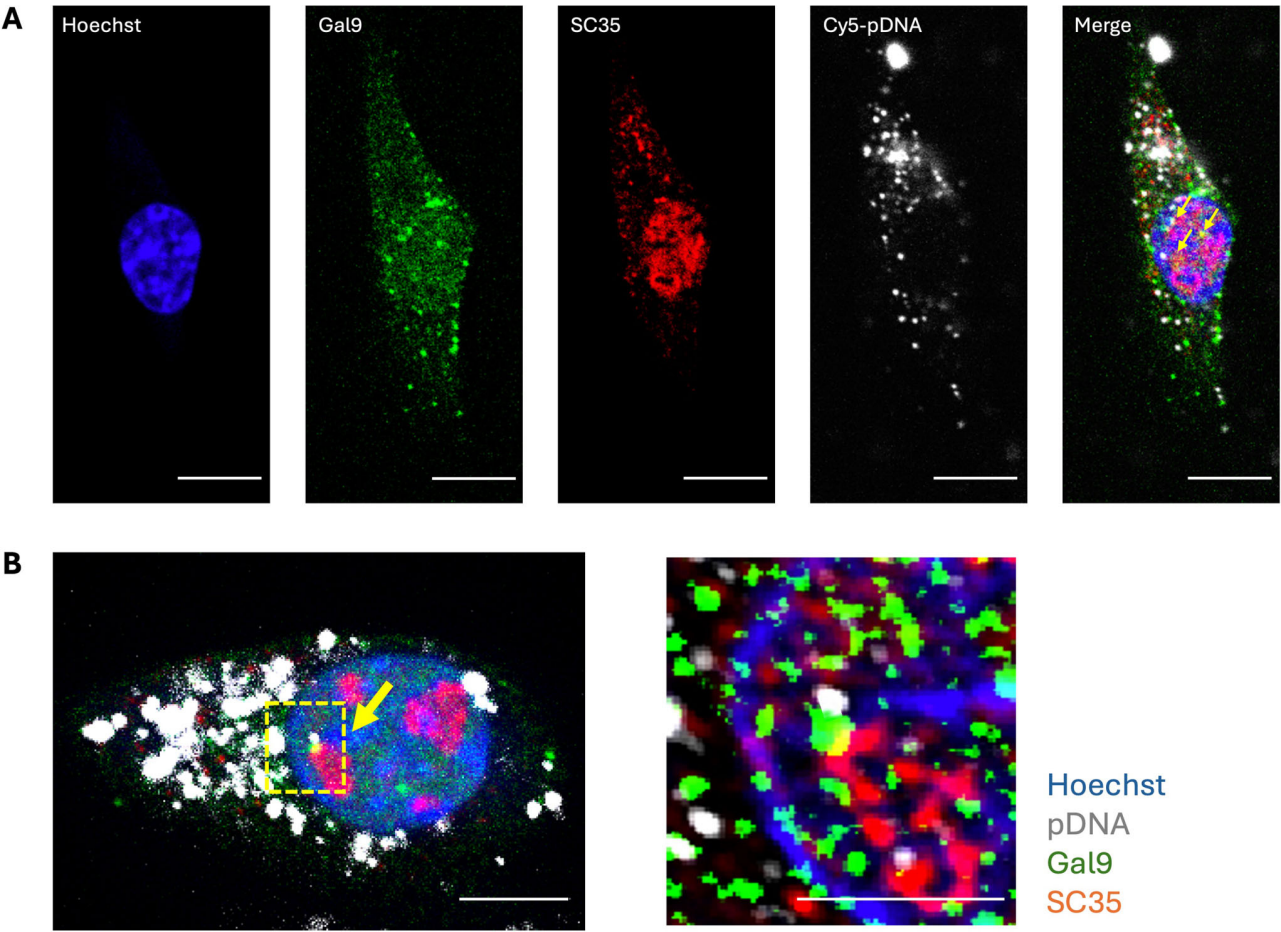
Galectin-9 colocalizes with transcriptional nuclear hubs and accumulates in speckle-like regions following pDNA electrotransfection. **(A)** Representative confocal fluorescence images of C2C12 cells electrotransfected with Cy5-labeled pDNA, acquired 1 h post-electroporation. Hoechst (blue) stains the nucleus, Gal9-GFP (green) shows Gal9 localization, SC35 (red) is detected using a specific antibody, and Cy5-pDNA is shown in white. The merged image highlights areas of colocalization between Gal9 and SC35 (yellow arrow). **(B)** Representative confocal image showing the association of Gal9, pDNA, and SC35 at 3 h post-electroporation (left panel). The yellow arrow indicates a triple colocalization among Gal9, SC35, and pDNA. The right panel shows a super-resolution enlargement of the boxed region from the left panel. Scale bars: 10 μm.

To explore this, we performed immunofluorescence analysis on Gal9-GFP expressing C2C12 cells after electrotransfection of pDNA. Cells were electrotransfected with Cy5-labeled pDNA, and at 1 or 3 h post-ET, stained with a fluorescently conjugated antibody targeting SC35, an essential pre-mRNA splicing factor and well-established marker of nuclear speckles involved in transcription and RNA processing. Confocal microscopy revealed that Gal9-GFP partially overlapped with SC35-positive speckles (Fig. 3A), and pDNA puncta were frequently observed in regions where both Gal9 and SC35 signals were present (Fig. 3B). The triple colocalization supports our hypothesis that Gal9 may contribute to the recruitment or retention of pDNA at nuclear speckles, the sites that likely serve as transcriptionally active hubs.

**Figure 4.**
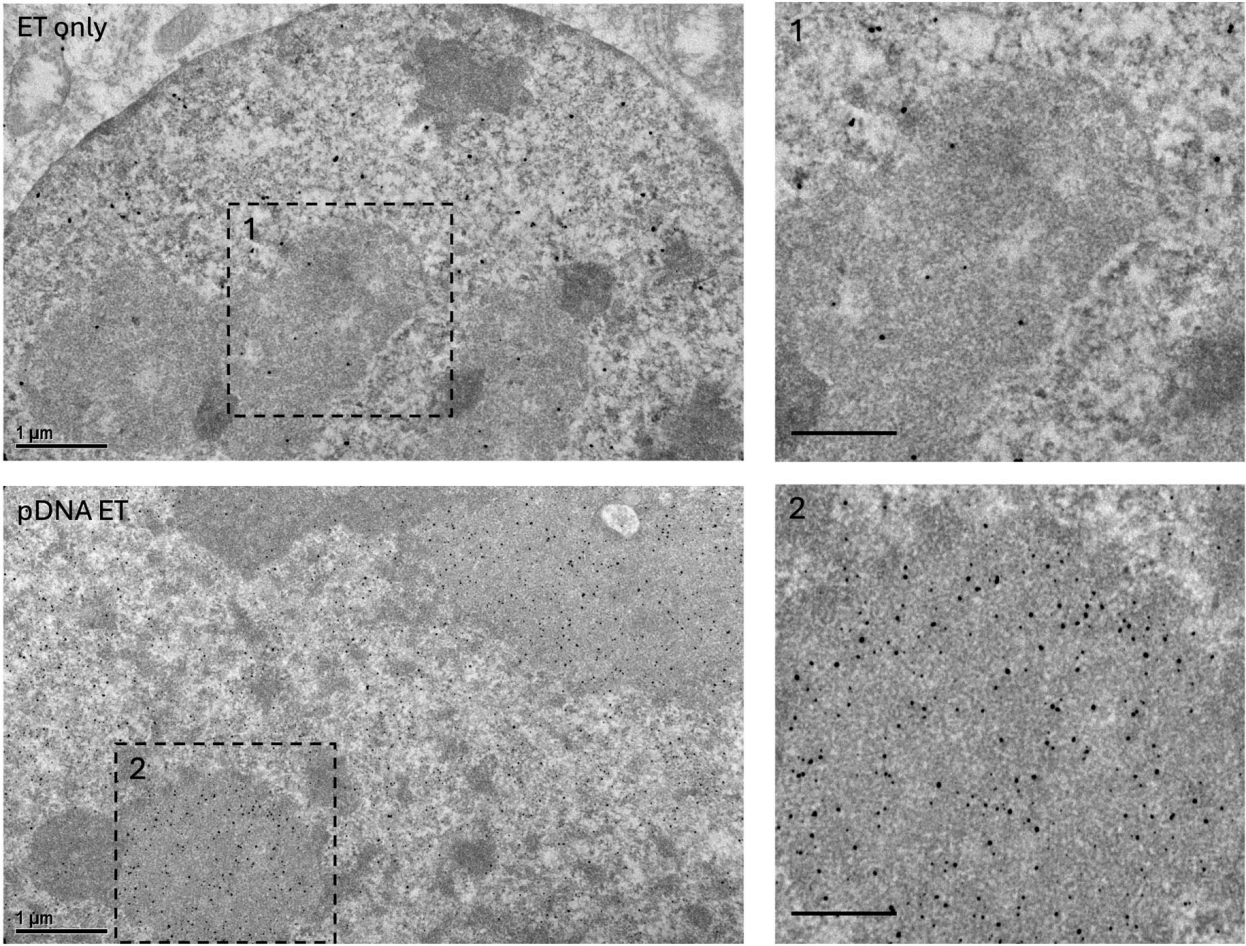
Immunogold TEM of C2C12 nuclei following electrotransfection with or without pDNA. Immunogold TEM images of C2C12 nuclei following pDNA electrotransfection (bottom) or electrotransfection without plasmid (ET only, top), acquired 6 h post-ET. Gold particles represent Gal9 signals labeled first by a primary anti-Gal9 antibody. Scale bars: 1 μm (overview images). Boxed regions are enlarged in the corresponding panels on the right. Scale bars: 200 nm (insets).

To further examine this association at higher resolution, we conducted immunogold transmission electron microscopy (immuno-TEM) using an antibody against Gal9. In cells fixed 6 h post-electrotransfection with pDNA, Gal9 labeling was enriched in dense subnuclear regions morphologically consistent with nuclear speckles and transcriptionally active chromatin domains (Fig. 4, bottom panels). In contrast, cells that underwent electrotransfection without pDNA (ET only) displayed minimal Gal9 accumulation in comparable nuclear areas (Fig. 4, top panels). These observations suggest that increased nuclear accumulation of Gal9 following pDNA delivery is not a result of the electric pulse, but rather reflects a functional role for Gal9 in facilitating pDNA trafficking and recruitment to transcriptionally active regions within the nucleus, consistent with the colocalization signals observed in confocal images.

Together, these findings indicate that Gal9 plays a role in the spatial organization of electrotransfected pDNA within the nucleus. Gal9 appears to associate with nuclear speckle subdomains, potentially facilitating the recruitment and stabilization of pDNA at sites of active transcription. The observation of sustained Gal9 nuclear signals at multiple time points post-electroporation further supports its involvement in ongoing transcriptional processes following pDNA delivery.

### Galectin-9-mediated DNA trafficking depends on delivery method and cargo type

To investigate whether Galectin-9 involvement in nucleic acid trafficking depends on both the delivery method and the cargo type, we examined its temporal colocalization with plasmid DNA following electrotransfection and Lipofectamine-mediated transfection, and further compared its association with different nucleic acid cargos, including plasmid DNA, minicircle DNA, and mRNA. This combined analysis allowed us to determine whether Gal9’s function is influenced by the route of cellular and nuclear entry as well as by the molecular characteristics of the delivered cargo.

**Figure 5.**
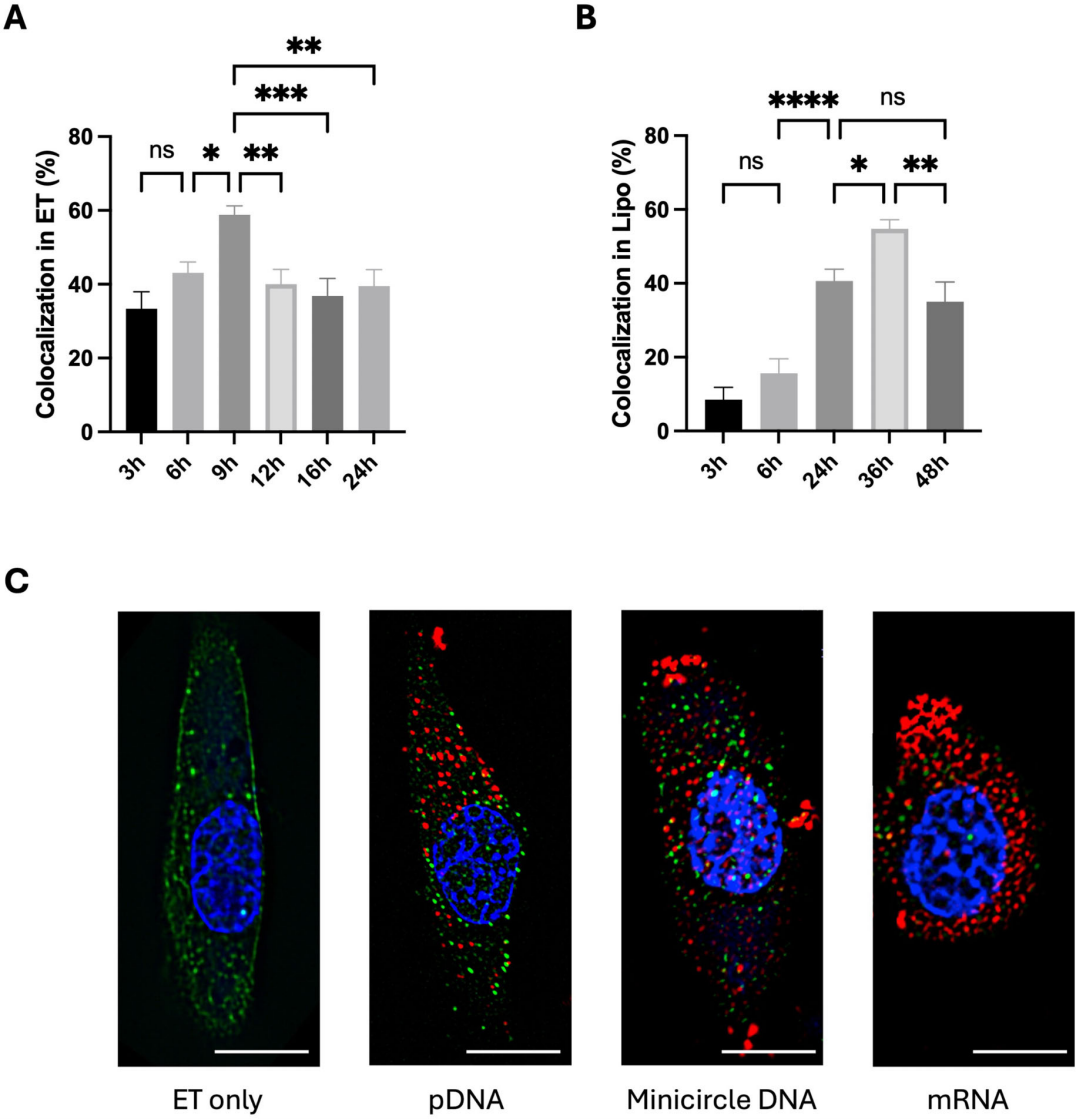
Time-dependent colocalization of Galectin-9 with plasmid DNA following electrotransfection and Lipofectamine-mediated transfection. **(A)** Quantification of Gal9-GFP and pDNA colocalization in C2C12 cells following electrotransfection. Cells transfected with pDNA were fixed at different time points (3, 6, 9, 12, 16, and 24 h) and analyzed by *in-situ* hybridization. **(B)** Quantification of Gal9-GFP and pDNA colocalization in cells transfected with Lipofectamine 3000. Cells were fixed at 3, 6, 24, 36, and 48 h post-transfection and assessed by *in-situ* hybridization. Data are shown as mean ± SEM (*n* = 15–25, each time point); Statistical analysis was performed using one-way ANOVA followed by Tukey’s HSD test. ns, not significant; *P < 0.05; **P < 0.01; ***P < 0.001; ****P < 0.0001. **(C)** Representative confocal images of Gal9-GFP expressing C2C12 cells electrotransfected with Cy5-labeled nucleic acid cargos. C2C12 cells were pulsed with pDNA, minicircle DNA, mRNA, or without cargo (ET only) and imaged by confocal fluorescence microscopy at 1 h post-ET. Confocal images showing Gal9 (green), nucleic acid cargos (red), and nuclei (blue). Scale bars: 10 μm.

Across both electrotransfection and lipofectamine delivery platforms, Gal9-pDNA colocalization was observed, but the timing and pattern of involvement differed significantly (Fig. 5). In electrotransfected cells, colocalization emerged early, with approximately 30% detected at 3 h and peaking at around 60% by 9 h post-transfection, before gradually declining and plateauing by 24 h (Fig. 5A). In contrast, Lipofectamine-mediated transfection exhibited delayed Gal9 involvement. Representative immunofluorescence images illustrating this delayed nuclear Gal9–pDNA association and colocalization with SC35-positive nuclear speckles following lipofectamine delivery are shown in Supplementary Fig. S3. Quantitatively, minimal colocalization was observed at early time points (3–6 h), with ∼8% and ∼15% respectively, but a substantial increase occurred at 24 and 36 h post-transfection, reaching a peak of ∼55% at 36 h, followed by a decrease at 48 h (Fig. 5B).

These results suggest that Gal9 is involved in pDNA nuclear trafficking and organization across both physical and chemical delivery methods, but the timing of its involvement is different depending on the delivery method. The earlier colocalization observed in electrotransfected cells likely reflects the faster intracellular kinetics of electrotransfection, where pDNA rapidly enters the nucleus and initiates transcription. In contrast, Lipofectamine-transfected cells require more time for the lipid–pDNA complexes to be internalized, followed by endosomal escape and nuclear entry. These findings indicate that Gal9 plays a role in intracellular pDNA processing and transcriptional activation, with the dynamics of its involvement varying with the delivery platform.

To further assess whether Gal9’s role depends on the nature of the nucleic acid cargo, cells were electrotransfected with plasmid DNA, minicircle DNA, mRNA, or pulsed in the absence of cargo (Fig. 5C). At 1 h post-electroporation, strong nuclear localization of both Gal9 and minicircle DNA was observed, resembling the pattern seen with plasmid DNA. In contrast, cells transfected with mRNA exhibited minimal nuclear Gal9 signal, and little to no mRNA was detected in the nucleus. In cells subjected to electric pulsing without nucleic acid cargo, Gal9 puncta remained largely cytoplasmic with minimal nuclear accumulation, indicating that Gal9 nuclear localization is not a generalized response to electrotransfection-associated stress but is closely associated with DNA trafficking. Consistent with this, Gal9 nuclear association is specific to DNA-based cargos such as plasmid DNA and minicircle DNA, rather than mRNA, which functions in the cytoplasm and does not undergo nuclear import. Moreover, minicircle DNA produced a greater number of nuclear puncta at early time points, which likely resulted from its smaller molecular size and higher efficiency of nuclear entry. This observation suggests that Gal9 facilitates or stabilizes DNA during nuclear transport, thereby promoting efficient transcriptional initiation once nuclear entry occurs.

Together, these findings demonstrate that Galectin-9 involvement in nucleic acid trafficking is both delivery method- and cargo-dependent. Gal9 facilitates the nuclear trafficking and early transcriptional engagement of DNA-based cargos across different transfection platforms, while RNA cargos, which function primarily in the cytoplasm, appear to bypass this Gal9-associated pathway.

### Galectin-9 expression modulates electrotransfection efficiency

To further assess the functional role of Galectin-9 in the electrotransfection of plasmid DNA, we evaluated the effects of both Gal9 knockdown and overexpression on reporter protein expression in C2C12 cells.

**Figure 6.**
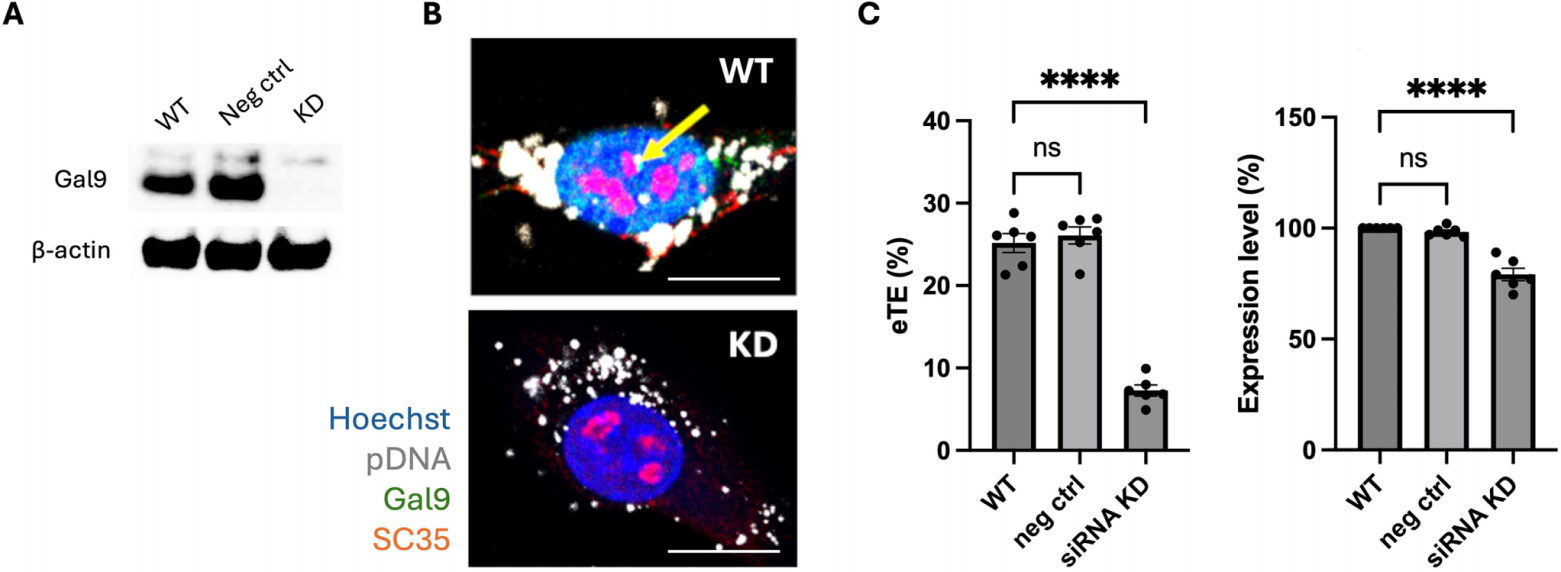
Knockdown of Galectin-9 reduces electrotransfection efficiency and pDNA transcription. **(A)** Western blot analysis confirming Gal9 knockdown in C2C12 cells treated with Gal9-specific siRNA compared to WT and negative siRNA (Neg) controls. β-actin was used as an internal control. **(B)** Representative confocal images showing nuclear distribution of Gal9, SC35, and pDNA in WT and Gal9 knockdown (KD) cells. Gal9-GFP (green), pDNA (white), SC35 (red), and nuclei stained with Hoechst (blue). The yellow arrow indicates colocalization of Gal9, pDNA, and SC35 in WT cells. Scale bars: 10 μm. **(C)** Quantification of electrotransfection efficiency (eTE, left) and EGFP reporter expression level (right) by flow cytometry in WT, negative control siRNA, and Gal9 siRNA knockdown groups. Data are shown as mean ± SEM (*n* = 6); one-way ANOVA followed by Tukey’s HSD test was performed: ****P < 0.0001, ns, not significant.

We performed siRNA-mediated knockdown of Gal9 and measured electrotransfection efficiency (eTE) and transgene expression following transfection with a plasmid encoding EGFP. Western blot analysis confirmed efficient Gal9 knockdown in siRNA-treated cells compared to untreated wild-type (WT) controls and negative siRNA controls (Fig. 6A). Confocal imaging showed a loss of Gal9 puncta and reduced intranuclear pDNA signals, along with diminished colocalization between pDNA and SC35-marked nuclear speckles in Gal9 knockdown cells (Fig. 6B). Quantitative flow cytometry analysis revealed that Gal9 knockdown led to approximately a 70% reduction in the percentage of EGFP-positive cells and a 20% decrease in EGFP expression level among the remaining positive cells, compared to both WT and negative control siRNA groups (Fig. 6C). These results indicated that Gal9 depletion primarily impaired the ability of cells to initiate productive pDNA transcription. Importantly, among the remaining cells that still expressed EGFP, fluorescence intensity per cell was relatively preserved, likely attributable to heterogeneous Gal9 knockdown, leaving a small expressing subpopulation. Together, these findings support an essential role for Gal9 in enabling efficient pDNA transcription following electrotransfection.

**Figure 7.**
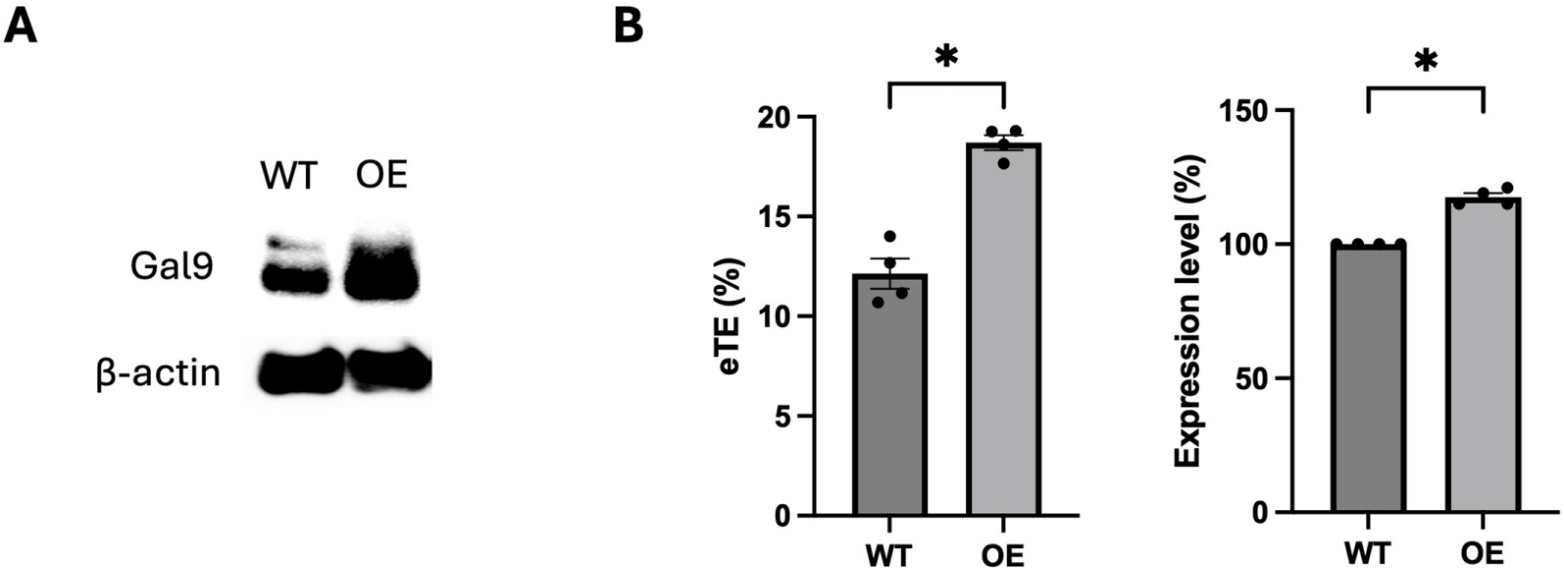
Overexpression of Galectin-9 enhances electrotransfection efficiency and transgene expression. **(A)** Western blot confirming Gal9 overexpression (OE) in C2C12 cells compared to wide type (WT) controls. β-actin served as an internal control. **(B)** Quantification of electrotransfection efficiency (eTE, left) and EGFP reporter expression level (right) by flow cytometry in WT and Gal9-overexpressing cells. Data are shown as mean ± SEM (*n* = 4); Mann–Whitney U test: *P < 0.05.

Conversely, overexpression of Gal9 significantly enhanced both electrotransfection efficiency and transgene expression (Fig. 7). Western blot analysis confirmed elevated Gal9 protein levels in the overexpression group compared to WT controls (Fig. 7**A**). Flow cytometry analysis showed that Gal9 overexpression increased eTE by ∼54% and enhanced EGFP expression level by ∼18% (Fig. 7B). These findings indicate that elevated Gal9 levels can effectively promote pDNA delivery and expression.

Together, these results demonstrate that Gal9 functionally modulates the efficiency of gene delivery and expression following electrotransfection, likely through its role in intranuclear trafficking and transcriptional recruitment of pDNA.

### Endogenous Galectin-9 level influences cell-type-dependent pDNA electrotransfection efficiency

**Figure 8.**
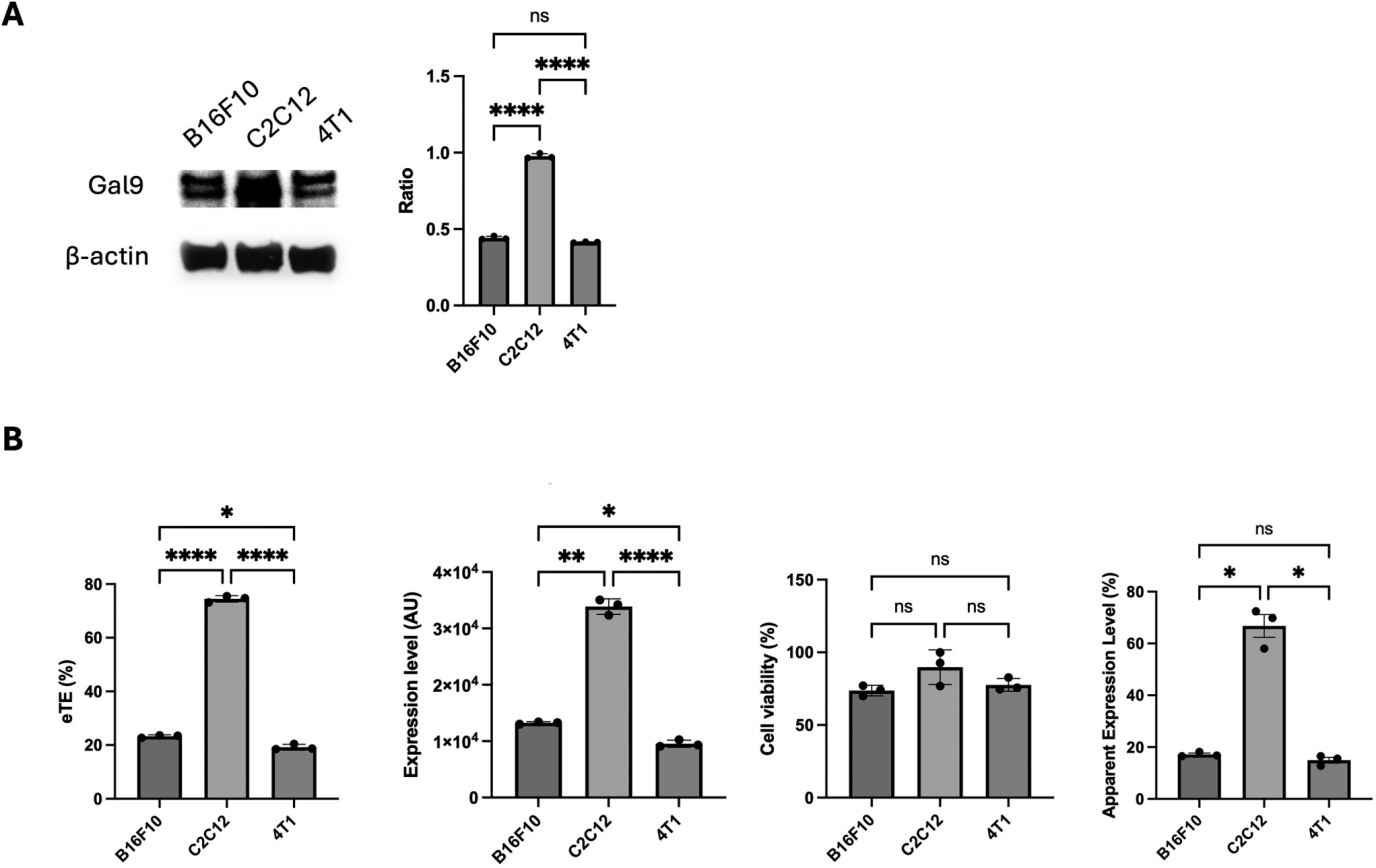
Correlation between Galectin-9 expression and pDNA electrotransfection efficacy across different cell lines. **(A)** Western blot analysis of Gal9 protein levels in B16F10, C2C12, and 4T1 cells, with β-actin used as a control. **(B)** Comparison of eTE, EGFP expression level, cell viability, and apparent expression level in cells electrotransfected with an EGFP-encoding plasmid. Data are shown as mean ± SEM (*n* = 3); one-way ANOVA followed by Tukey’s HSD test was performed: *P < 0.05; **P < 0.01; ***P < 0.001; ****P < 0.0001, ns, not significant.

Electrotransfection efficiency often varies among cell types, reflecting differences in membrane properties, intracellular trafficking, and gene expression machinery. To investigate whether Galectin-9 expression contributes to these differences, we compared its basal protein levels and pDNA electrotransfection outcomes across three representative cell lines: C2C12 myoblasts, B16F10 melanoma cells, and 4T1 breast cancer cells (Fig. 8). Internal Gal9 protein levels were analyzed in cell lines by western blot. Gal9 expression was markedly higher in C2C12 cells, approximately 2.2-fold higher than in B16F10 and 2.4-fold higher than in 4T1 cells (Fig. 8A). This finding indicates that Gal9 expression is cell type-dependent and may be influenced by tissue origin or differentiation state, consistent with prior studies showing that Gal9 levels vary across epithelial, myogenic, and tumor-derived cells due to differences in transcriptional regulation and intracellular stress signaling [2, 14].

To assess whether these differences in Gal9 abundance relate to gene delivery efficiency, the same cell lines were electrotransfected with a plasmid encoding EGFP. Consistent with the expression pattern of Gal9, C2C12 cells exhibited substantially higher electrotransfection efficiency (eTE) and transgene expression level compared with B16F10 and 4T1 cells (Fig. 8B). Cell viability remained comparable across groups, indicating that the variation in transfection performance was not due to cytotoxicity. When compared cohesively as an apparent expression level (the product of eTE, expression level, and viability), C2C12 cells displayed the highest overall outcome, approximately 3.9-fold and 4.5-fold greater than B16F10 and 4T1 cells, respectively. These results suggest that higher endogenous Gal9 expression levels enhance electrotransfection-mediated pDNA delivery and expression, likely by promoting more efficient intracellular trafficking and nuclear entry of pDNA. In contrast, tumor-derived cell lines such as B16F10 and 4T1, which express lower levels of Gal9, may have reduced capacity to support Gal9-associated pDNA transport into the nucleus, leading to lower electrotransfection performance.

## Discussion

The current study identified a previously unrecognized nuclear role of Galectin-9 in the intracellular transcriptional processing of plasmid DNA following non-viral gene delivery. Previous studies have characterized Gal9 as a cytosolic sensor of damaged endosomal and lysosomal membranes, where it contributes to innate immune activation and inflammatory signaling during lipid-based nucleic acid delivery, including liposomes and lipid nanoparticles [15, 16]. In these contexts, Gal9 recruitment to disrupted vesicles has been linked to cytokine production and delivery-associated toxicity, and inhibition of Gal9 signaling has been explored as a strategy to mitigate inflammation and improve the safety and efficacy of certain nucleic acid–based therapeutics [17, 18]. Although Gal9 is well known for its role as a reporter of damaged endosomes and lysosomes, we observed a distinct and unexpected localization of Gal9 within the nucleus following electrotransfection of pDNA. This intranuclear accumulation and involvement with pDNA were observed in a time-dependent manner and were particularly enriched in pDNA-positive cells, suggesting a direct association between Gal9 and the delivered DNA cargo. The presence of Gal9 as punctate in nuclear structures beyond its canonical cytoplasmic distribution expands our understanding of its function in intracellular trafficking and raises new questions about its involvement in transcriptional regulation.

Mechanistically, our findings suggest that Gal9 may facilitate the recruitment of pDNA to transcriptionally active nuclear domains. Through immunofluorescence and immuno-TEM analyses, we demonstrated that Gal9 colocalizes with SC35-marked nuclear speckles, the subnuclear hubs enriched in transcriptional and RNA-processing machinery. Furthermore, Gal9, pDNA, and SC35 were found to colocalize as discrete nuclear foci following electrotransfection. This observation is consistent with our previous findings that punctate nuclear pDNA structures are more transcriptionally engaged than diffuse pDNA signals [13]. It also aligns with prior literature studies showing that pDNA molecules recruited to transcription hubs or co-transcriptional splicing form dense nuclear hubs visualized as foci in cell nuclei [19–21]. These results collectively support a mechanism in which Gal9 may guide or retain pDNA at transcriptional hotspots in the nucleus, enhancing the probability of productive gene expression.

We also found that Gal9’s involvement in pDNA delivery is not limited to electrotransfection. A time-dependent comparison of Gal9-pDNA colocalization between two delivery platforms, electrotransfection and Lipofectamine-mediated transfection, revealed different temporal patterns. In electrotransfected cells, Gal9 localization in the nucleus alongside pDNA occurred rapidly after pulsing, consistent with the known fast kinetics of nuclear pDNA accumulation and transcriptional activation via electrotransfection, with detectable protein expression as early as 6 h post-ET. This observation also aligns with our previous study quantifying the copy number of intranuclear pDNA post-electrotransfection over time [22], supporting a consistent role for Gal9 in nuclear pDNA transcriptional processing. In contrast, Lipofectamine-mediated delivery showed delayed Gal9 recruitment to the nucleus, likely due to the additional time required for endocytic uptake, endosomal escape, and nuclear import of pDNA either as the lipid-pDNA complexes or as free pDNA after dissociation from the lipid. Notably, Gal9 puncta were also observed in the cytoplasm following electrotransfection, consistent with vesicular engagement prior to nuclear entry. Although electrotransfection is initiated by electric pulsing, accumulating evidence indicates that internalized plasmid DNA subsequently engages endocytic and endolysosomal trafficking pathways [9, 10]. These observations suggest that Gal9 participates in vesicular sensing or trafficking events prior to nuclear accumulation, regardless of the gene delivery method used, and that the differences observed between electrotransfection and Lipofectamine-mediated delivery in both the duration of Gal9 engagement at vesicular membranes and the timing of its subsequent nuclear accumulation may reflect delivery method-dependent differences in endosomal stress magnitude and escape kinetics.

Moreover, the cargo-dependent pattern of Gal9 involvement further supports its role as a selective mediator of DNA trafficking. The absence of Gal9 nuclear localization with mRNA delivery underscores that its function is tied to processes unique to DNA-based cargos, particularly those requiring nuclear entry and engagement with intranuclear transcriptional machinery. The faster and stronger Gal9 association observed with minicircle DNA suggests that smaller, more transcriptionally accessible DNA molecules may be recruited more efficiently to the transcriptionally active nuclear domains. Together, these findings indicate that Gal9 contributes to organizing exogenous DNA within transcriptionally favorable nuclear microenvironments, where both the delivery route and cargo structure influence the kinetics and extent of its involvement.

In addition to the confocal imaging–based observations of Gal9 involvement, functional modulation of Gal9 expression levels further demonstrated its regulatory role in electrotransfection effectiveness. Knockdown of Gal9 significantly reduced both electrotransfection efficiency and transgene expression levels, whereas overexpression of Gal9 enhanced both metrics. These functional assays underscore the importance of Gal9 not only in pDNA intranuclear localization but also in transcriptional output. The data suggest that Gal9 may serve as a critical mediator of nuclear pDNA dynamics and activity, potentially functioning as a scaffold or adaptor protein that anchors plasmids to transcriptionally active regions within the nucleus.

The cell-type-dependent differences in Gal9 expression and electrotransfection efficacy highlight its importance in regulating cellular responsiveness to non-viral DNA delivery. The elevated Gal9 level in C2C12 myoblasts may be linked to the high membrane dynamics and fusion activity inherent to muscle differentiation, where other galectins such as Galectin-1 and Galectin-3 have been shown to promote adhesion and fusion processes [23–25]. Such functions could indirectly enhance the intracellular trafficking and nuclear engagement of electrotransfected DNA. In contrast, the lower Gal9 expression typically observed in tumor-derived cells, reported to aid immune evasion and resistance to apoptosis [1, 26, 27], may limit their capacity for efficient DNA transport and transcriptional activation. These findings suggest that endogenous Gal9 abundance shapes the cellular environment for productive pDNA electrotransfection.

Collectively, our findings reveal a novel nuclear function of Galectin-9 in modulating gene delivery efficiency. Beyond its established role in sensing endosomal and lysosomal membrane damage, Gal9 contributes to the spatial regulation of plasmid DNA within the nucleus and supports transcriptional activity. These insights suggest potential new mechanisms in non-viral gene delivery and highlight Gal9 as a candidate for future investigation in gene transfer optimization. Future studies may explore whether modulating Gal9 or related nuclear trafficking factors could enhance delivery efficiency across various cell types and platforms, and inform the identification of small molecules or biologics that improve nucleic acid delivery.

## Supplementary Material

ugag020_Supplemental_File

## Data Availability

The data that support the findings of this study are available from the corresponding author, F.Y., upon reasonable request.
