# Supplementary material for: Functional role of Galectin-9 in nucleic acid trafficking and transcription post-electrotransfection
Source: NAR Mol Med. 2026 Mar 26;3(2):ugag020. doi: 10.1093/narmme/ugag020 (PMC13069676; doi:10.1093/narmme/ugag020)
Supplement: ugag020_Supplemental_File [file ugag020_supplemental_file.pdf]

Supplementary Materials

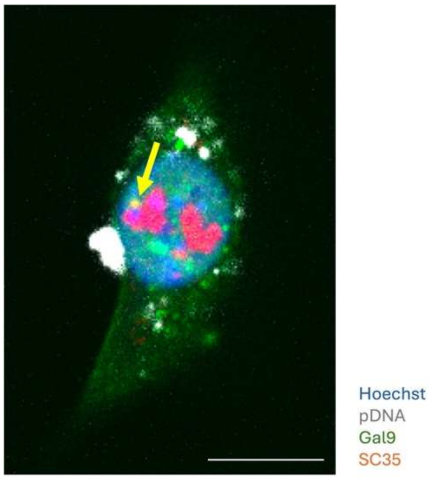

Figure S1. Representative confocal fluorescence image of C2C12 cells electrotransfected with Cy5-labeled plasmid DNA (pDNA, white) and immunostained for endogenous Galectin-9 (green) and the nuclear speckle marker SC35 (red) at 3 hours post-electrotransfection. Nucleus was counterstained with Hoechst (blue). The image demonstrated that endogenous Galectin-9 accumulated within the nucleus and partially colocalized with SC35-positive transcriptional domains (yellow arrow), consistent with observations obtained using the GFP-Gal9 fusion construct. Scale bar: 10 μm.

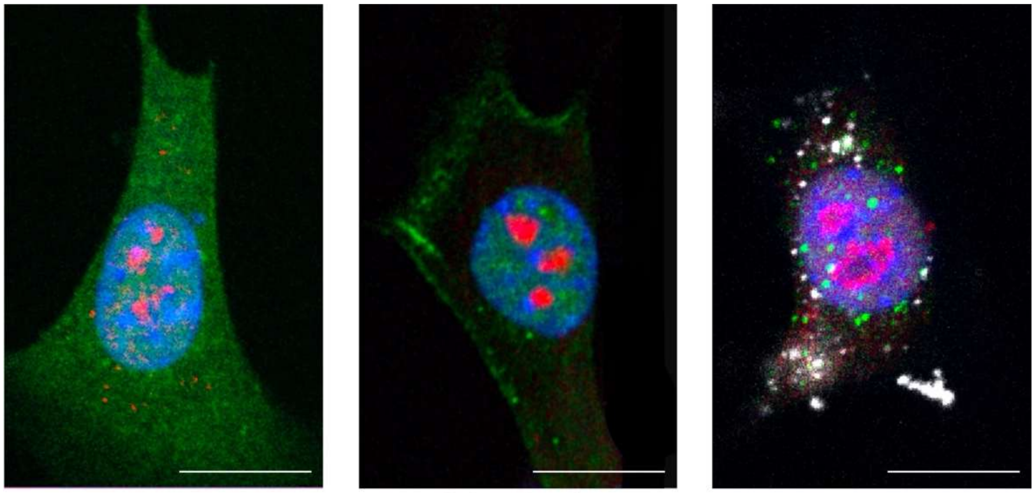

Figure S2. Representative confocal fluorescence images of C2C12 cells under three conditions. Left: non-transfected control cells without electric pulsing or plasmid DNA, showing predominantly diffuse and homogeneous Galectin-9 (green) distribution. Middle: pulse-only control cells subjected to electric pulsing in the absence of nucleic acid cargo, displaying limited Galectin-9 puncta primarily localized in the cytoplasm periphery with minimal nuclear accumulation at 1 hour post ET. Right: cells imaged at 1 hour post-ET with plasmid DNA, exhibiting pronounced redistribution of Galectin-9 into distinct punctate structures with substantial nuclear accumulation. Nuclear speckles were labeled by SC35 (red), and nuclei were counterstained with Hoechst (blue). These images demonstrated that Galectin-9 puncta formation and nuclear accumulation were dependent on plasmid DNA electrotransfection. Scale bars: 10 μm.

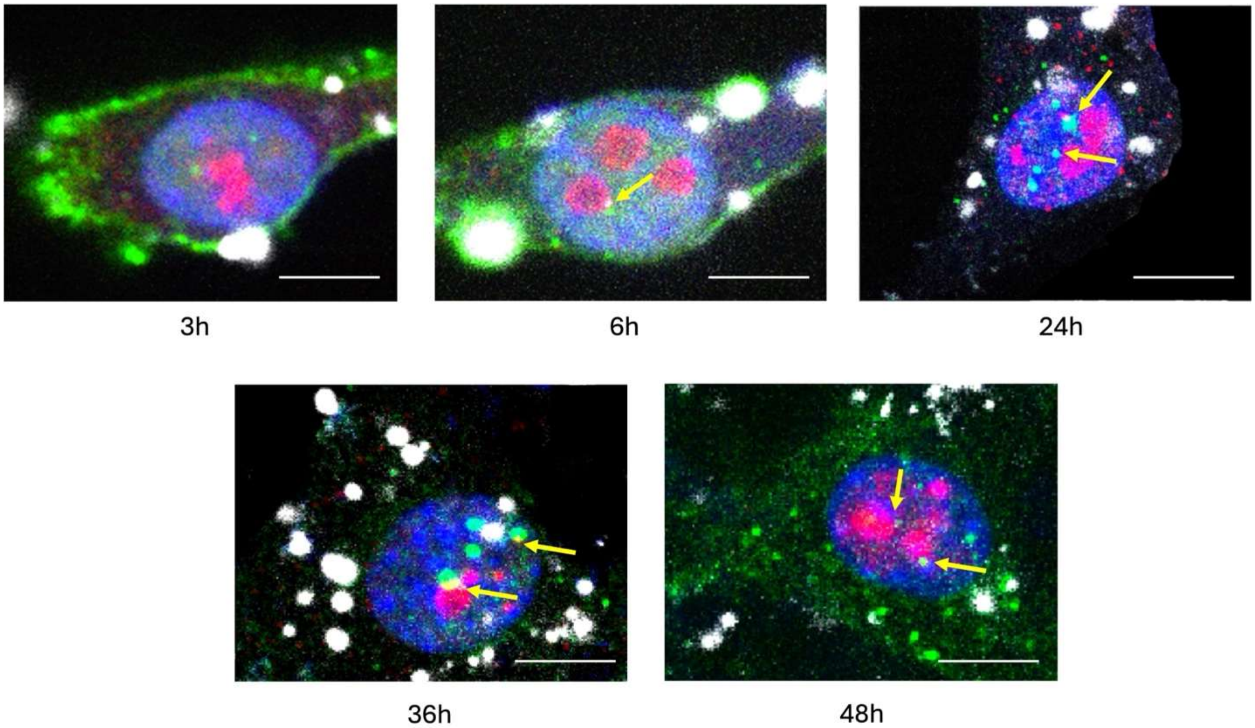

Figure S3. Representative confocal fluorescence images of C2C12 cells transfected with Cy5-labeled plasmid DNA using Lipofectamine and imaged at 3, 6, 24, 36, and 48 hours post-transfection. Galectin-9 is shown in green, plasmid DNA in white, nuclear speckles labeled by SC35 in red, and nuclei counterstained with Hoechst in blue. Yellow arrows indicate representative nuclear regions where Galectin-9 puncta colocalize with plasmid DNA. some of which are associated SC35-positive nuclear speckles. The images illustrate delayed yet pronounced nuclear Gal9–pDNA association compared to that observed in electrotransfection experiments. Scale bars: 10 μm.
